# Metagenomic Analysis Reveals the Response of Microbial Communities and Their Functions in Lake Sediment to Environmental Factors

**DOI:** 10.3390/ijerph192416870

**Published:** 2022-12-15

**Authors:** Dan Song, Tangbin Huo, Zhao Zhang, Lei Cheng, Le Wang, Kun Ming, Hui Liu, Mengsha Li, Xue Du

**Affiliations:** 1 Heilongjiang River Fisheries Research Institute, Chinese Academy of Fishery Sciences, Harbin 150070, China; 2 Heilongjiang River Basin Fisheries Ecology Observation and Research Station of Heilongjiang Province, Harbin 150070, China; 3 College of Animal Science and Technology, Northwest A&F University, Yangling 712100, China; 4 A Reserve Assets Authority, Harbin 150030, China; 5 Institute of Nature and Ecology, Heilongjiang Academy of Sciences, Harbin 150040, China

**Keywords:** metagenomics, lake sediments, microbial community, metabolic functions, Jingpo Lake

## Abstract

Jingpo Lake is the largest mountain barrier lake in China and plays a key role in breeding, power generation, and providing a source of drinking water. Microbes are important participants in the formation of lake resources and energy cycles. However, the ecological protection of Jingpo Lake has faced serious challenges in recent years. In this study, we investigate the responses of the microbial community’s composition of sediments at five locations to an environmental gradient representing water quality and water-depth changes using a metagenomic sequence. We found that the diversity and composition of the microbiota sediments were altered spatially and correlated with the physicochemical factors of water samples. In the microbial community, relatively lower Chao1, alternating conditional expectations, and Shannon and Simpson indices were found at the shallowest location with higher total phosphorus and chlorophyll *a*. Furthermore, the Kyoto Encyclopedia of Genes and Genomes analysis revealed that the metabolism function was the most abundant functional classification in Jingpo Lake. The levels of total phosphorus, chlorophyll *a* and pH were positively correlated with the abundance of *Flavobacterium* and the bacterial functions of the carbohydrate metabolism and amino acid metabolism. In conclusion, our results reveal the physical and chemical characteristics, as well as the microbial community characteristics, of Jingpo Lake, which provides new insights for studying the relationship between environmental factors and the bacterial community distribution of freshwater ecosystems, in addition to also providing a theoretical basis for the environmental monitoring and protection of the lake.

## 1. Introduction

Land-use change is transforming freshwater ecosystems; elevated allochthonous inputs of nutrients and organic compounds to freshwaters have contributed significantly to the accelerated levels of eutrophication and pollution [1,2], altering the community structure and functions of aquatic biota. Global climate change has attracted widespread attention due to its potential impact on natural ecosystems [3,4,5]. The global average temperature rose by about 0.89 °C during the 20th century. In the next fifty years, the global average temperature will rise by about 2–4 °C [6]. Further climate change will likely have more pronounced effects in temperate regions, and it may interact with other anthropogenic impacts, such as land-use change, for example, by increasing water temperatures or by changing stratification patterns, affecting nutrient loads in lake catchments [7,8]. Multistress factors will produce interactions that are additive, synergistic or antagonistic [9]. Lakes are an important type of inland water body, accounting for about 83% of the total area of inland water bodies. Meanwhile, lakes are also the connection point for the interaction between water, soil and gas in the Earth surface system, and have a significant impact on the global and regional material element cycles [10,11]. Therefore, variations in water quality parameters and nutrient cycling, largely resulting from climate and land-use changes, are a chief environmental focus in aquatic ecosystems [12,13].

The microorganism is an important component of the aquatic environment, including bacteria, archaea and eukaryotes. Microorganisms play an important role in the geochemical cycle. As decomposers and primary producers, microorganisms not only participate in the material cycling of lake water, but also provide important energy and material sources for the lake through the decomposition of some animals, plants and organic matter, such as through carbon cycling, nitrogen cycling and the transformation of some trace elements [14,15]. Therefore, the study of lake microorganisms is very significant for revealing the element cycling process of lake ecosystems and its response mechanism to environmental changes, as well as for deeply understanding the structure and function of lake ecosystems. The microbial community composition in sediments always changes with environmental changes, and thus, is often used as a biological indicator in the lake environment. Previous studies have showed that environmental variables, such as organic matter [16], nutrient concentration [17], salinity [18], pH [19], heavy metals [20] and macrophyte identity [21], have been proven to be important factors influencing the bacterial diversity and community structure in lake sediments. Microbial communities are expected to dominantly consist of generalist species, rather than specialist species, as they are well-adapted to the eutrophic environment [16,22]. For example, dechlorinating and contaminant-degrading bacteria were positively correlated with the excessive accumulation of organic matter and nutrients in lake sediments [23,24,25]. Moreover, in the sediments of eutrophic lakes, microorganisms are expected to progressively adapt their metabolic mechanisms to carry C-, N- and P-cycle-related genes for the removal of excess C, N and P [26].

Jingpo Lake is the largest mountain barrier lake in China. It has a prominent position and important economic benefits because of its role in freshwater aquaculture, tourism, power generation and providing a source of drinking water [27,28]. In recent years, due to the impact of global climate change and human activities, the ecological protection of Jingpo Lake has faced serious challenges. With climate warming and enhanced anthropogenic activities within the Jingpo Lake watershed, including urbanization, deforestation for agricultural development, tourism and hydrological alteration, the lake has faced the risk of serious pollution from nitrogen, phosphorus and heavy metals due to industrial waste water, agricultural waste water and domestic sewage [29,30,31]. As an important part of lake ecosystems, microorganisms drive the cycling of nutrient elements and the migration and transformation of pollutants in the lake [32,33]. At the same time, the community composition and function of microorganisms are also affected by the element contents and environmental factors. As aquatic ecosystems are already responding to land-use change at the watershed scale [34], and these land-use-related changes are expected to increase with climate change [35,36], it is necessary to gather data on the current state of the microbial community and its functions in Jingpo Lake to provide a theoretical basis for lake protection and research in the future.

In this study, we characterized the microbial community composition and diversity in the sediment of Jingpo Lake and revealed the microbial community structure’s response to environmental parameters using metagenomics. The study also sought to examine the bacterial functional profiles and to demonstrate the effects of physiochemical factors on microbial function. We hypothesized that the taxonomic composition and diversity of the bacterial community depend strongly on eutrophic-related factors, and that differences in the functions of the bacterial community are determined by the taxonomic composition.

## 2. Materials and Methods

### 2.1. Study Site Description and Sampling Procedure

**Figure 1:**
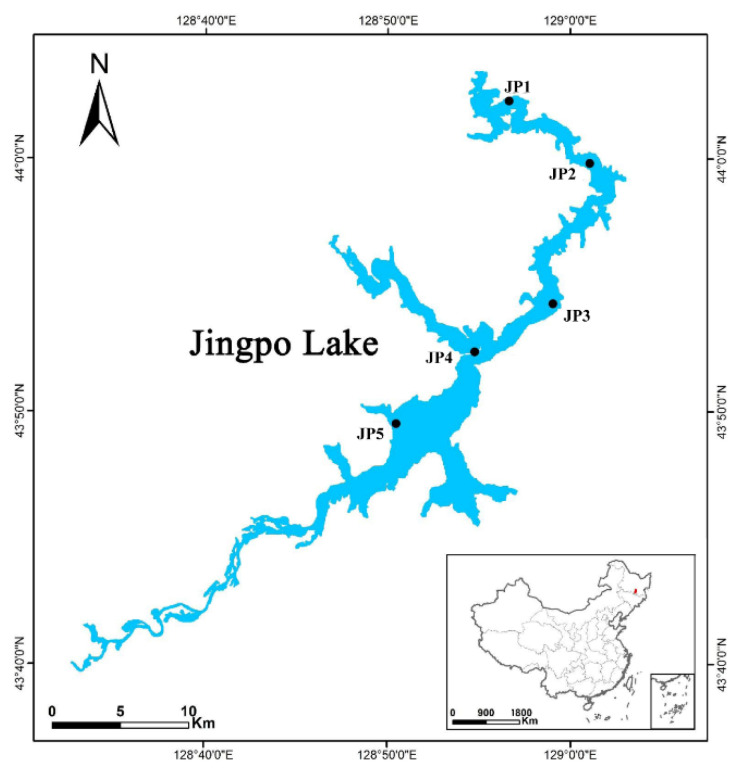
Sampling sites of Jingpo Lake.

This study was conducted in Jingpo (Figure 1), the largest mountain barrier lake located in Northeastern China (43°46′–44°18′ N, 120°30′–129°30′ E). Jingpo Lake has an area of 90.3 km^2^, with a maximum length and width of 45 and 6 km, respectively. The average depth is about 40 m. Jingpo is normally covered in ice from October to April, with an average annual air temperature of 2.6 °C. Surface water and sediments were collected from five locations of Jingpo Lake, namely, JP1, JP2, JP3, JP4 and JP5 (Figure 1), in May 2021. We sampled the top ~40 cm of sediment with a gravity corer (Uwitec Ltd., Mondsee, Austria). The sediment samples were placed into sterile centrifuge tubes and immediately stored at −80 °C until needed for further analysis.

Concurrent with the sediment sampling, 5 L of the 15 L pooled water chemistry samples were collected at each sampling location. We also measured water depth (WD) with a handheld depth finder, and water temperature (WT), dissolved oxygen (DO), electrical conductivity (EC) and pH with a portable YSI Professional Plus instrument (YSI Incorporated, Yellow Springs). Other chemical variables, including total phosphorus (TP), total nitrogen (TN), ammonia nitrogen (AN), nitrite nitrogen (NIN), nitrate nitrogen (NAN), permanganate (PG) and chlorophyll *a* (Chl *a*), were measured in accordance with the standard methods [37].

### 2.2. DNA Extraction and Metagenomic Sequencing

Genomic DNA from the JP1, JP2, JP3, JP4 and JP5 groups was extracted using HiPure Soil DNA Kits (Magen, Guangzhou, China), according to the manufacturer’s instructions. Firstly, the sediment samples were homogenized and treated with lysis buffers. Subsequently, the homogenate cleavage of sediments was carried out by utilizing the MP FastPrep-24 (USA) to fully lyse the samples, followed by incubation at 75 °C for 10 min. Afterwards, the sample was purified by employing a HiPure DNA Mini Column II (Magen, Guangzhou, China) to conduct the centrifuging and washing steps, following the manufacturer’s instructions. Finally, the DNA was eluted with elution buffers. The DNA quality was determined using the Qubit fluorometer (Thermo Fisher Scientific, Waltham, MA, USA) and the Nanodrop spectrophotometer (Thermo Fisher Scientific, Waltham, MA, USA).

Qualified genomic DNA was fragmented to a size of 350 bp by sonication, and subsequently, end-repair, A-tail and adaptor ligation were conducted using the NEBNext^®^ Ultra™ DNA Library Prep Kit for Illumina (NEB, Ipswich, MA, USA), following the preparation protocol. DNA fragments with a length of 300–400 bp were enriched using PCR. Finally, PCR products were purified using the AMPure XP system (Beckman Coulter, Brea, CA, USA), and libraries were analyzed for size distribution by using the 2100 Bioanalyzer (Agilent, Santa Clara, CA, USA) and were quantified via a real-time PCR. Genome sequencing was carried out on the Illumina Novaseq 6000 sequencer via pair-end technology (PE 150).

### 2.3. Bioinformatic Analysis

Raw data were filtered using FASTP (version 0.18.0) with the following standards: (1) removing reads with more than 10% of nucleotides (N); (2) removing reads with more than 50% of bases having Q quality scores less than 20; and (3) removing reads containing adapters. After filtering, the obtained clean reads were used for genome assembly.

Clean reads of each sample were assembled using MEGAHIT (version 1.1.2). Genes were predicted based on the final assembly contigs (>500 bp) using MetaGeneMark (version 3.38). The predicted genes that were more than 300 bp in length from all samples were pooled and combined on the basis of a ≥95% identity and 90% read coverage using CD-HIT (version 4.6). The reads were realigned to the initial non-redundant gene set using Bowtie (version 2.2.5). Based on the comparison results, the reads were reassigned to the best genes using the PathoScope software. Genes with ≤2 reads in each sample were filtered out to obtain the final gene set for subsequent analysis.

We used several complementary approaches to annotate the assembled sequences. The unigenes were annotated using DIAMOND (version 0.9.24) by aligning them with the deposited ones in different protein databases, including the National Center for Biotechnology Information (NCBI) non-redundant protein database (Nr, https://www.ncbi.nlm.nih.gov/refseq/; accessed on 26 May 2022), and the Kyoto Encyclopedia of Genes and Genomes (KEGG, http://www.genome.jp/kegg/; accessed on 26 May 2022).

### 2.4. Statistical Analysis

The reads were aligned with the Nr microbial library (including bacteria, fungi, archaea, viruses, microfauna and plants) for species annotation using Kaiju software (version 1.6.3). The differences in read abundances for the specific phyla and genera were identified by using STAMP software. We used Mothur (http://www.mothur.org/wiki/Calculators; accessed on 26 May 2022) to calculate the alpha diversity indices. Differences in alpha diversity among sites were identified using the Kruskal–Wallis test. The Bray–Curtis distance matrix based on the relative functional abundance and the taxonomic abundance was determined by using the R 4.0.3 [38] *vegan* package [39]. To analyze functional and taxonomic composition structures of sediment metagenomes, a principal coordinate analysis (PCoA) was conducted based on Bray–Curtis distances using the R *vegan* package [39]. A Venn diagram was plotted to show the genera present in all samples of a site and those shared among sites. Biomarker features in each group were screened by using a linear discriminant analysis, carried out via the effect size (LEfSe) software (version 1.0). The redundancy analysis (RDA) was used to determine the effects of water physicochemical variables on microbial composition and function in Jingpo Lake using the *vegan* package [39]. The graphs were drawn and analyzed using GraphPad Prism 7.0 (GraphPad Software, Inc., San Diego, CA, USA) and R 4.0.3 [38]. A *p*-value < 0.05 was considered statistically significant.

## 3. Results

### 3.1. Physicochemical Characteristics of the Sampling Sites

The physical and chemical measurements from Jingpo Lake are presented in Table 1. Water temperature, dissolved oxygen, electrical conductivity, pH, total phosphorus and chlorophyll *a* were all the highest at JP5. Meanwhile, nitrate and total nitrogen were the highest at JP1, whereas they were the lowest at JP5.

### 3.2. Microbial Community Composition

**Figure 2:**
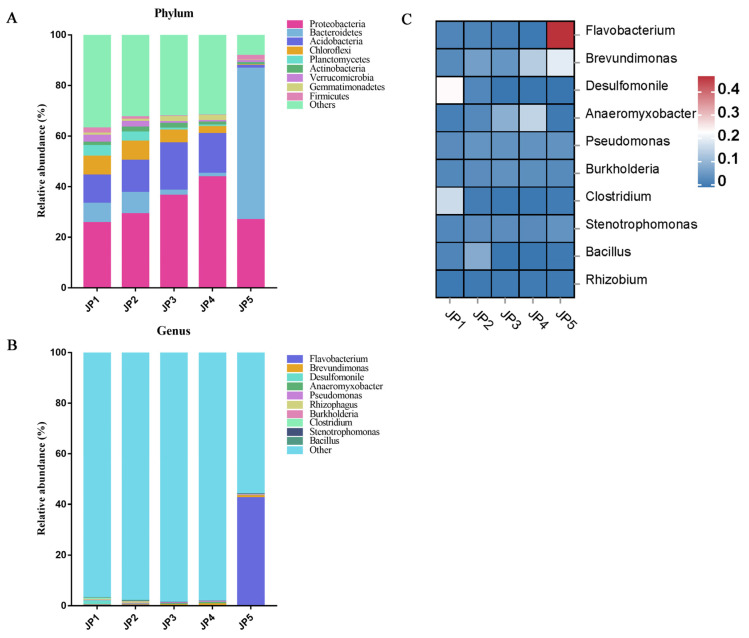
Microbial community composition of sediment at five different locations of Jingpo Lake. Phylum (**A**) and genus (**B**) abundance comparison among five different regions of Jingpo Lake. (**C**) Heatmap of differentially abundant bacterial genera. Color scale represents the row-scaled, log-transformed relative abundances of genera.

The top 10 most abundant bacteria taxa are displayed in Figure 2. In this study, the bacterial structure of all groups was analyzed at the phylum level and the genus level. At the phylum level, the main taxa included *Proteobacteria*, *Bacteroidetes*, *Acidobacteria*, *Chloroflexi*, *Planctomycetes*, *Actinobacteria* and *Verrucomicrobia*. There was no differentially abundant phylum among JP1, JP2, JP3 and JP4, except *Proteobacteria*. With the decrease in water depth, the relative abundance of *Proteobacteria* increased from 25.63% to 43.79%. In addition, the microbial community composition of JP5 changed dramatically. The relative abundance of *Bacteroidetes* at JP5 (59.76%) was highest compared with JP1, JP2, JP3 and JP4. In contrast, the relative abundance of *Acidobacteria* (1.13%) was lowest at JP5. At the genus level, the degree of difference among the groups was similar to that at the phylum level. The bacterial composition was very similar for JP1, JP2, JP3 and JP4. The main genus at JP1, JP2, JP3 and JP4 included *Brevundimonas* and *Desulfomonile*. Nevertheless, *Flavobacterium* (46.69%) was the most dominant genus at JP5.

### 3.3. Differences in Microbial Communities of Sampling Sites

**Figure 3:**
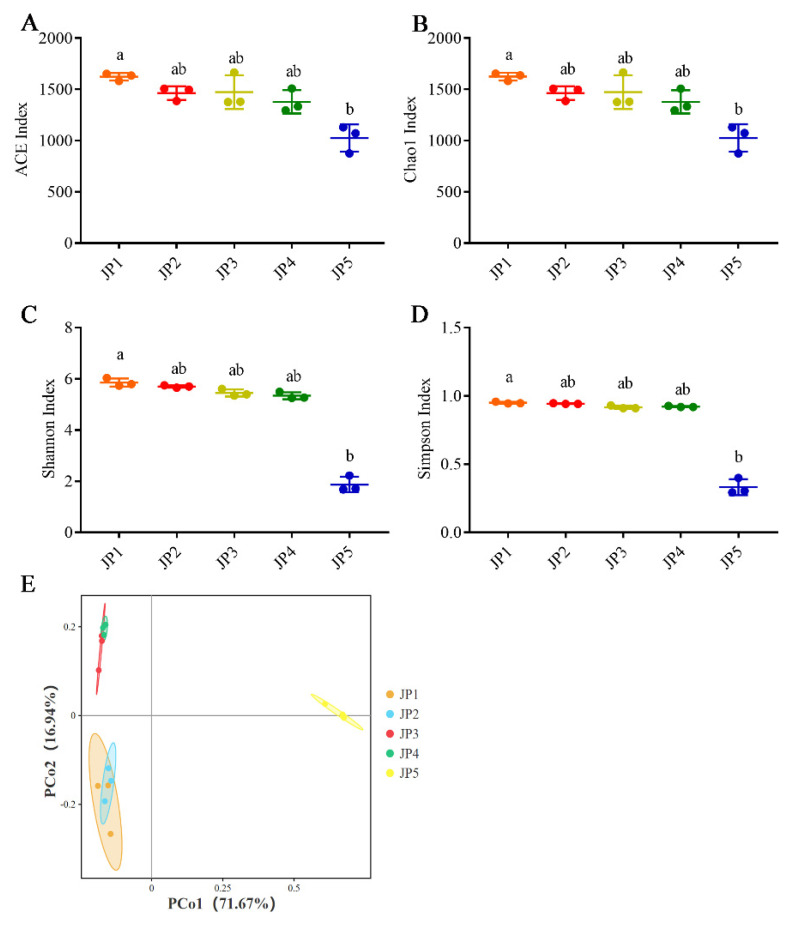
Alpha diversity and beta analysis based on sediments in Jingpo Lake. (**A**) ACE. (**B**) Chao1. (**C**) Shannon. (**D**) Simpson. (**E**) Principal coordinate analysis (PCoA). Statistical analyses of alpha diversity were performed using Kruskal−Wallis test. ^a,b^ Bars marked with different letters indicate significant differences (*p* < 0.05). PCoA analysis was performed based on the Bray−Curtis matrix.

We analyzed the differences in the sediment microbiome from five sampling sites. Regarding microbiota community diversity, a significant difference was observed in various alpha-diversity indices between JP1 and JP5 (*p* < 0.05, Kruskal–Wallis test) (Figure 3). Additionally, the principal coordinate (PCoA) analysis based on the Bray–Curtis dissimilarities of these bacterial communities indicated that there were significant differences among different sampling sites (*p* = 0.001, *r* = 0.7941, ADOSIM) (Figure 3E). It is worth noting that there was a much greater marked difference between the bacterial structure from JP5 and the other four sampling sites.

**Figure 4:**
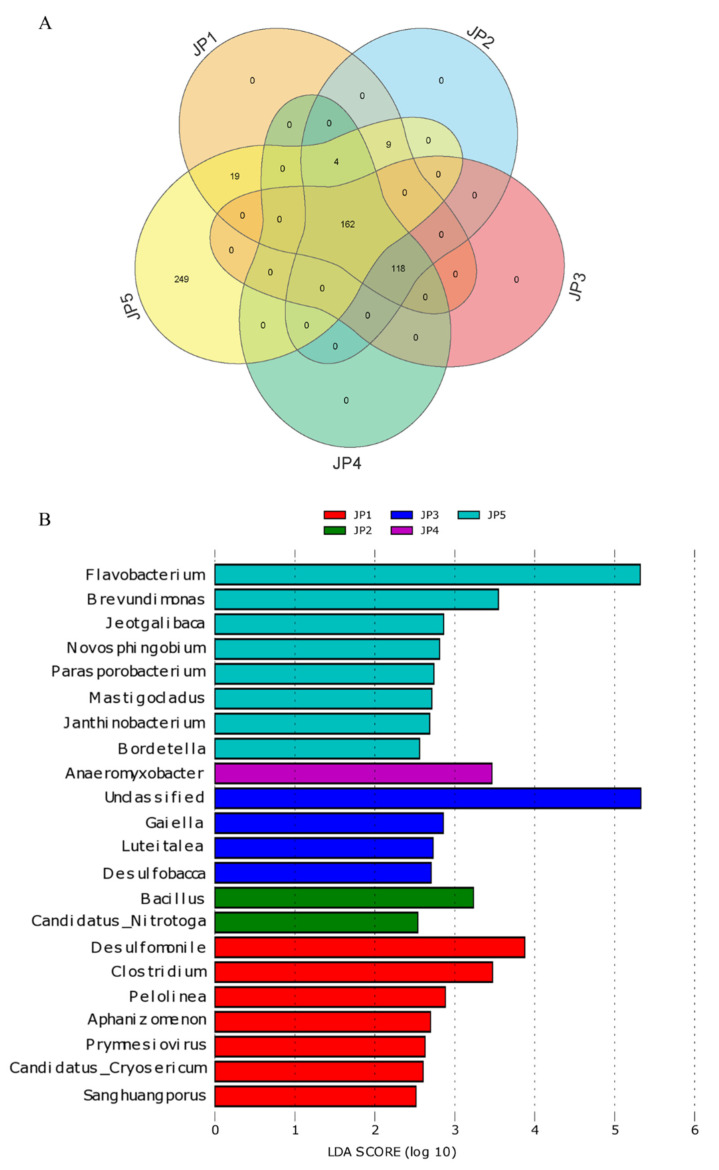
Differences in the abundances of bacterial communities of sediments at five different locations of Jingpo Lake. (**A**) Venn diagram of genera present in all samples of a group and those shared among groups. (**B**) The log-linear discriminant analysis (LDA) effect size quantifies the degree to which each lineage contributes to the uniqueness of each group. Statistical analyses of LEfSe were performed using Kruskal–Wallis test and Wilcoxon rank-sum test.

There were 443 conserved genera present at JP5, 162 of which were shared with all other samples (JP1, JP2, JP3 and JP4), whereas 249 unique genera were found at JP5 (Figure 4A). In contrast, no unique genera were conserved at JP1, JP2, JP3 and JP4. To further analyze the bacteria with statistically significant differences among all sampling sites, the LDA effect size (LEfSe) method was used for comparison in this study (Figure 4B). At the genus level, we found that *Flavobacterium* was the biomarker at JP5. Simultaneously, the proportion of *Flavobacterium* at JP5 was significantly higher than for the other four groups, which was confirmed by Tukey’s multiple comparison tests (*p* < 0.01). At JP4, we found that *Anaeromyxobacter* was the most abundant taxon. *Bacillus* and *Desulfomonile* were significant biomarkers at JP2 and JP1, respectively.

### 3.4. Differences in the KEGG Function of Sampling Sites

**Figure 5:**
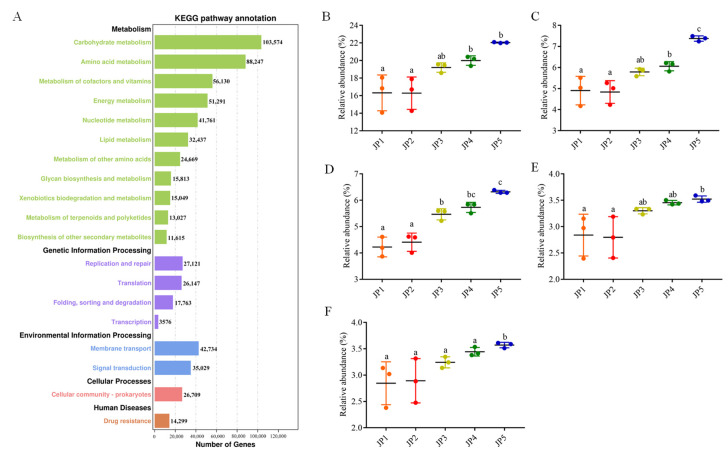
Differences in the KEGG function of all sampling sites. (**A**) KEGG function annotation of unigenes for metagenome sequencing of Jingpo Lake. Comparison of the abundance of microbial KEGG function among five different regions of Jingpo Lake. (**B**) Metabolism; (**C**) carbohydrate metabolism; (**D**) amino acid metabolism; (**E**) metabolism of cofactors and vitamins; (**F**) energy metabolism. Color scale represents the row-scaled, log-transformed relative abundances of function. Statistical analyses of alpha diversity were performed using Kruskal−Wallis test. ^a,b,c^ Bars marked with different letters indicate significant differences (*p* < 0.05).

Based on metagenomic sequencing, 1,490,487, 1,536,083, 1,419,634, 1,249,434 and 817,084 contigs were obtained from the JP1, JP2, JP3, JP4 and JP5 groups, respectively. All unigenes were annotated using KEGG databases. The results showed that the annotation success rate of unigenes was 1,507,922 in the KEGG databases (73.12%). The level of metabolism showed significant differences among the five samples (*p* < 0.01, ordinary one-way ANOVA) and significantly increased for JP4 and JP5 (*p* < 0.01, Tukey’s multiple comparison test) (Figure 5). From the KEGG databases, 1,507,922 unigenes were classified into 19 functional categories. Among them, carbohydrate metabolism (103,574 unigenes), amino acid metabolism (88,247 unigenes), the metabolism of cofactors and vitamins (56,130 unigenes) and energy metabolism (51,291 unigenes) were the top four KEGG pathways (Figure 5). Meanwhile, the top four KEGG pathways all displayed significantly higher contributions at JP5 (Figure 5).

### 3.5. Correlations between Physicochemical Factors, Microbial Community and KEGG Metabolism Function

**Figure 6:**
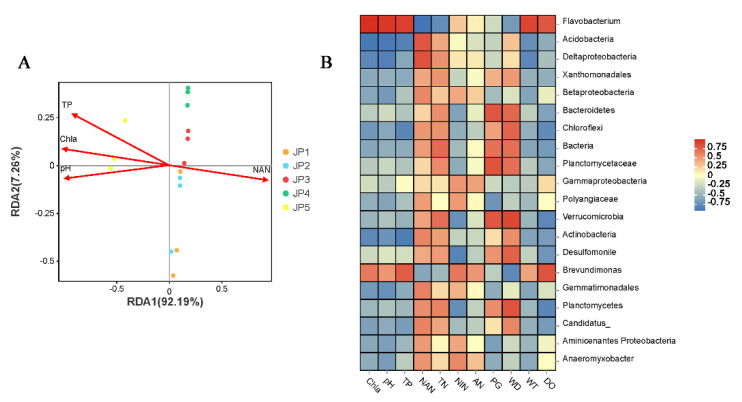
Correlation between physicochemical factors and microbial genera. (**A**) Redundancy analysis (RDA) depicted the relationship between the top four environmental indicators and the composition of water microbiota among five groups. (**B**) Correlation heatmap between microbial abundance and all physicochemical factors. Statistical analyses of correlations were performed using Pearson correlation analysis.

**Figure A1:**
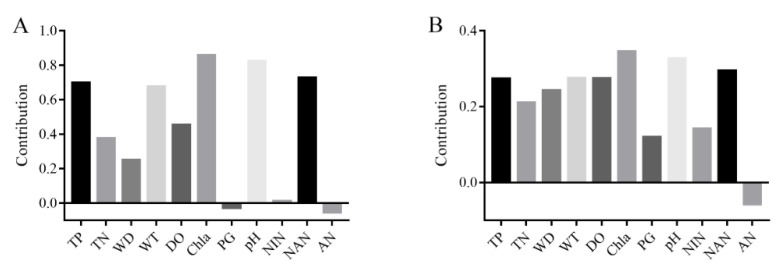
Contribution of physicochemical factors to the bacterial community composition (**A**) and function (**B**). Statistical analyses were performed using variance partitioning analysis.

The redundancy analysis (RDA) was used to determine the extent of physicochemical factors affecting the microbial composition through a detrended correspondence analysis (DCA) (Table A1). The top four environmental indicators (Chl *a*, pH, NAN and TP) were selected for RDA analysis (Figure A1A). The first axis explained 92.19% and the second axis explained 7.26% of the variation in the RDA biplot (Figure 6A). Our study revealed a considerable correlation between sediment microbiota and physicochemical factors. The levels of Chl *a*, pH and TP were all positively correlated with JP5, whereas the level of NAN was negatively correlated with JP5. At the genus taxonomy level, *Flavobacterium* showed a strong positive correlation with Chl *a*, pH and TP, and had a negative correlation with NAN (Figure 6B). Meanwhile, there was an opposite trend of correlation between *Acidobacteria* and the four physicochemical indicators.

**Figure 7:**
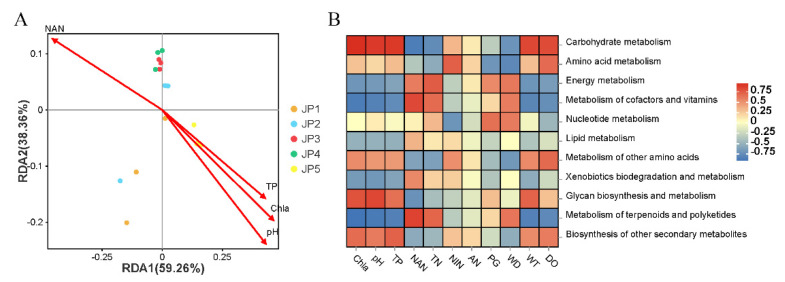
Correlation between physicochemical factors and microbial metabolism functions. (**A**) Redundancy analysis (RDA) depicted the relationship between the top four environmental indicators and the level B microbial metabolism functions among five groups. (**B**) Correlation heatmap between level B microbial metabolism functions and all physicochemical factors. Statistical analyses of correlations were performed using Pearson correlation analysis.

The RDA analysis was used to determine the extent of physicochemical factors affecting microbial functions through a DCA analysis. In order to be consistent with the microbial composition, the top four environmental indicators (Chl *a*, pH, NAN and TP) were selected for RDA analysis (Figure A1B). The first axis explained 59.26% and the second axis explained 38.36% of the variation in the RDA biplot (Figure 7A). Similar to the results for bacterial composition, the levels of Chl *a*, pH and TP were all positively correlated with JP5, whereas the level of NAN was negatively correlated with JP5. For the top four KEGG functions of level B, the carbohydrate metabolism and amino acid metabolism both showed a strong positive correlation with Chl *a*, pH and TP and had a negative correlation with NAN. In contrast, the metabolism of cofactors and vitamins and energy metabolism both displayed a strong negative correlation with Chl *a*, pH and TP and had a positive correlation with NAN (Figure 7B).

## 4. Discussion

The interaction between water, land material and energy forms the abundant natural resources in lakes and provides the most basic material sources for the survival and reproduction of the people living in the lake area. In recent years, with the development of industrialization, the lake environment has deteriorated to different degrees, and lake health has become a hot research topic [40,41]. Jingpo Lake, as the second largest mountain barrier lake in the world, greatly contributes to water storage, power generation, fishing and hunting, and tourism [42]. At present, Jingpo Lake has begun to show eutrophication [43]. Therefore, the inspection of the water quality of Jingpo Lake is very important. In this study, we carried out a metagenomic analysis to understand the relationship between the physicochemical factors and sediment microbiota of Jingpo Lake.

Previous studies have shown that the composition and species diversity of lake microbial communities were related to temperature, dissolved oxygen, light intensity, pH, salinity and other environmental factors [44]. In this study, the physicochemical parameters of JP5 showed great differences compared to the other four groups. The Chl *a* and pH levels of the JP5 group in Jingpo Lake were higher than that of the JP1 group, which we speculate to be related to the biological activities of algae and plankton. Total nitrogen (TN) and total phosphorus (TP) are important environmental factors affecting phytoplankton growth and reproduction. The average TN and TP of JP5 were 1.046 and 0.081 mg/L, which exceed the category “III” standard of 1.0 and 0.05 mg/L of China (GB3838-2002), respectively. Moreover, according to the trophic level index (TLI) [45], the TLI of JP5 was higher than 50 (data not displayed), suggesting a light eutrophication level. We discovered that the TN content decreased, whereas TP content increased in the JP5 group compared with the JP1 group. It is speculated that an increase in temperature may increase the growth rate of algae, which increases the consumption of nitrogen by organisms, resulting in a decrease in the concentration of total nitrogen. Increased total phosphorus concentrations in the JP5 group may be related to an endogenous release from river input and rainfall. Oueriaghli et al. [46] performed a study of Rambla Salada, a high-salt environment in Southeastern Spain and found that salinity and oxygen were the main environmental factors affecting bacterial communities. Lau et al. [47] conducted a study of Temenggor Lake in Malaysia and found that microbial communities had a certain dependence on light intensity. Results from 30 Wisconsin lakes indicated that pH was one of the most important factors in altering microbial communities [48].

Considering that JP5 is closer to shallow water than the other groups, human activity may have a greater impact in this area. Therefore, the bacterial community composition of JP5 is more likely to change. Our research observed that the alpha diversity at JP5 was lower than that of other groups and showed a significantly low level compared with JP1. These results illustrate that the unique physicochemical conditions of JP5 decreased the richness and evenness of the sediment microbiota. Therefore, compared with the other groups, the micro-ecological environment of JP5 is more unstable. Moreover, a significant difference was also observed in the beta diversity. These data all demonstrated that the bacterial community structure of JP5 was quite different from that of the other groups. As a typical mountain lake reservoir, Jingpo Lake has the characteristic of a single habitat structure [49]. Additionally, the biomass and diversity of macrophytes in Jingpo Lake have decreased in recent years [50]. The above situation may have led to the unique microbial community structure of JP5.

Subsequently, we analyzed the differences in the sediment microbiome at each sampling site. For all the sites, the common abundant microorganisms in Jingpo Lake were Proteobacteria, which is similar to the findings of many other freshwater lake studies [51,52]. However, Bacteroidetes was the most dominant in the JP5 group. As an important genus of Bacteroidetes, *Flavobacterium* had an absolute advantage in JP5. Evidence has shown that *Flavobacterium* was often discovered in a high abundance in eutrophic and hypertrophic urban rivers [53]. The genus *Flavobacterium* was reported to be associated with harmful algal blooms because it can lyse cyanobacteria cells [54] and degrade cyanobacterial toxins or other complex organic molecules [55,56]. Further research has revealed that a positive correlation exists between the concentrations of Chl *a* and the abundance of *Flavobacterium* in Jingpo Lake. In previous studies, it was also well-documented that Chl *a* has a strong correlation with *Flavobacterium* [57,58]. A similar trend occurs with pH and TP. Members of this genus also have nitrogen-fixing capacities and promote the growth and total nitrogen content of maize plants [59]. Therefore, these microorganisms play an important role in environmental governance and detection.

Numerous studies have shown that carbohydrate metabolism, amino acid metabolism and energy metabolism are all related to the main functions of bacteria [60,61]. From the point of view of the function of microbiota, we found that the most abundant functional classification was metabolism, of which the top four functions were carbohydrate metabolism, amino acid metabolism, the metabolism of cofactors and vitamins, and energy metabolism. In previous studies, many aquatic habitats have also been shown to exhibit a similar functional pattern as that found in our research [62,63,64]. The bacterial community composition was changed in a eutrophic state, leading to the significant upregulation of metabolic function genes of organic carbon and amino acids. Our results demonstrate that the top four functions of the JP5 group all displayed a higher level among the five groups. In particular, the abundance of carbohydrate metabolism and amino acid metabolism was significantly higher for JP5 than in the other four groups, indicating a more eutrophic state of JP5 compared to the other sites. *Flavobacterium*, the largest family of the *Bacteroidetes* phylum, are heterotrophs that utilize complex carbohydrates and proteins due to the high frequency and diversity of genes involved in the utilization of peptides, proteins and the metabolism of carbohydrates [65,66,67]. For example, *Flavobacteria* had a relatively large complement to both carbohydrate-active enzymes (CAZymes) and peptidases, playing an important role in the mineralization of organic matter [64]. These functions were known to play huge roles in bacterial biofilm formation and bacterial structural functions [68,69]. On the whole, the shallower area of Jingpo Lake showed the highest biological activity.

Considering that environmental physicochemical factors greatly affect the composition of microorganisms, as a result, the abundance of these functions is also directly related to physical and chemical factors [70]. Our results showed that the levels of TP, Chl *a* and pH were positively correlated with the bacterial function of JP5. Interestingly, carbohydrate metabolism and amino acid metabolism were both positively correlated with TP, Chl *a* and pH, but the metabolism of cofactors and vitamins and energy metabolism showed the opposite trend. Therefore, the influence of these differences in physicochemical factors on the microecological structure is mainly reflected in their positive interference with the carbohydrate metabolism and amino acid metabolism.

## 5. Conclusions

In conclusion, the physical and chemical properties of water were spatially different in Jingpo Lake. The differences in the microbial community composition and function from five different sites of Jingpo Lake were revealed in this study. Significant differences were found for the microbial characteristics and physicochemical indices of JP5 compared with the other four groups. These differences remind us of the instability of microecosystems at JP5. Considering that JP5 is closer to the areas of human activity, this may shed light on the influence of human factors on water quality. Therefore, the regulation of the dominant bacteria, *Flavobacterium*, in the JP5 area may contribute to improving the water environment. However, more studies are required to clarify the specific functions of *Flavobacterium* in the microecosystems of Jingpo Lake.

## Figures and Tables

**Table 1:** Physical and chemical variables of the water samples from the five sites of Jingpo Lake.

Parameters	JP1	JP2	JP3	JP4	JP5
Water depth (m)	41.6	30.2	21.7	12.4	5.0
Water temperature (°C)	14.5	14.7	15.3	13.9	16.9
Dissolved oxygen (mg/L)	9.1	10.1	10.2	10.9	12.1
Electrical conductivity (μs/cm)	96.1	93	91	91.6	102.8
pH	7.71	7.68	7.7	7.48	8.35
Total phosphorus (mg/L)	0.047	0.043	0.036	0.055	0.081
Total nitrogen (mg/L)	1.504	1.454	1.165	1.464	1.046
Ammonia (mg/L)	0.084	0.093	0.076	0.102	0.091
Nitrite (mg/L)	0.045	0.049	0.049	0.053	0.051
Nitrate (mg/L)	1.004	0.808	0.874	1.000	0.479
Permanganate (mg/L)	6.04	6.26	4.36	4.93	5.02
Chlorophyll *a* (mg/L)	13.10	12.77	11.93	9.45	48.37

## Data Availability

The raw metagenomic data (BioProject number: PRJNA889691) were deposited in the NCBI Sequence Read Archive database.

## Appendix A

**Table A1:** Results of detrended correspondence analysis.

Parameters	JP1	JP2	JP3	JP4
Eigenvalues	0.3721	0.1083	0.0114	0.0362
Decorana values	0.4139	0.0354	0.0038	0.0016
Axis lengths	2.3403	1.1780	0.8193	0.7493
